# Multicentre MDR *Elizabethkingia anophelis* isolates: Novel random amplified polymorphic DNA with capillary electrophoresis systems to rapid molecular typing compared to genomic epidemiology analysis

**DOI:** 10.1038/s41598-019-38819-w

**Published:** 2019-02-12

**Authors:** Ming-Jr Jian, Cherng-Lih Perng, Jun-Ren Sun, Yun-Hsiang Cheng, Hsing-Yi Chung, Yu-Hsuan Cheng, Shih-Yi Lee, Shu-Chen Kuo, Hung-Sheng Shang

**Affiliations:** 1 Graduate Institute of Medical Science, National Defense Medical Center, Taipei, Taiwan; 2 Division of Clinical Pathology, Department of Pathology, Tri-Service General Hospital, National Defense Medical Center, Taipei, Taiwan; 3 Institute of Preventive Medicine, National Defense Medical Center, Taipei, Taiwan; 4 Division of Clinical Microbiology, Department of Pathology and Laboratory Medicine, Taipei Veterans General Hospital, Taipei, Taiwan; 5 National Institute of Infectious Diseases and Vaccinology, National Health Research Institutes, Zhunan, Taiwan

## Abstract

*Elizabethkingia* species are ubiquitous bacteria that uncommonly cause human infection. *Elizabethkingia anophelis* was first identified in 2011 from the mosquito *Anopheles gambiae*. The currently available bacterial typing systems vary greatly with respect to labour, cost, reliability, and ability to discriminate among bacterial strains. Polymerase chain reaction (PCR)-based fingerprinting using random amplified polymorphic DNA (RAPD) is commonly used to identify genetic markers. To our knowledge, no system coupling RAPD-PCR and capillary gel electrophoresis (CGE) has been utilized for the epidemiological typing of *E. anophelis*. Thus, the aim of the present study was to establish a reliable and reproducible molecular typing technique for *E. anophelis* isolates based on a multi-centre assessment of bacteraemia patients. Here, we used a rapid CGE-light-emitting diode-induced fluorescence (LEDIF)-based method in conjunction with RAPD-PCR to genotype *E. anophelis* with a high level of discrimination. All clinical isolates of *E. anophelis* were found to be typeable, and isolates from two hospitals formed two distinct clusters. The results demonstrated the potential of coupling RAPD and CGE as a rapid and efficient molecular typing tool, providing a reliable method for surveillance and epidemiological investigations of bacterial infections. The proposed method shows promise as a novel, cost-effective, high-throughput, first-pass typing method.

## Introduction

The genus *Elizabethkingia* was named in honour of Elizabeth King, who first identified the bacterium in association with meningitis in infants. The genus comprises several species, including *Elizabethkingia miricola, Elizabethkingia meningoseptica*, and *Elizabethkingia anopheli*s. *E. anophelis*, previously known as *Chryseobacterium meningosepticum*, is widely distributed in soil and water. *E. anophelis* was previously identified as the causative agent of human disease in the Central African Republic, Singapore, and Hong Kong. Recently, a multistate cluster of *E. anophelis* infections was reported in the midwestern United States isolated from dozens of patients, and these infections were associated with significant morbidity and mortality^1–8^. *E. anophelis* is an emerging opportunistic pathogen, and *E. anophelis* infections are associated with a high mortality rate because of its resistance to multiple antibiotics. Therefore, *E. anophelis* infections should be considered a clinically significant problem, and investigation of antibiotic resistance mechanisms is warranted^9^.

Very little information is currently known regarding the routes of transmission of *E. anophelis* infections^9^. Nosocomial outbreaks of infection with *E. anophelis* have been reported in both neonatal and adult intensive care units^5,7,10^. The confirmed number of cases in the United States 2015–2016 outbreak of *E. anophelis* was 65, in which 20 people died from the infection; all the cases were associated with a similar strain^6^.

However, few studies have focused on the epidemiological typing of *E. anophelis*^9^. Fingerprinting techniques are important tools in understanding the genetic diversity of bacterial isolates and for the epidemiologic monitoring of outbreak-related situations^11,12^. A wide variety of bacterial typing systems are currently available, which show substantial variations with respect to the effort required, cost, reliability, and ability to discriminate among bacterial strains. No single technique is optimal for all forms of investigation. Random amplified polymorphic DNA (RAPD) analysis is based on the presence of primer-binding sites in the genome that are sufficiently similar to permit PCR amplification using a single primer with an arbitrary nucleotide sequence at a low annealing temperature. RAPD typing has proven to be useful for the epidemiological typing of bacterial isolates from human outbreaks.

Although multilocus sequence typing (MLST) and pulsed-field gel electrophoresis (PFGE) are powerful techniques for epidemiological typing, the random amplified polymorphic DNA (RAPD) PCR technique can rapidly process large amounts of strains in a relatively uncomplicated protocol^11^, and is commonly applied to detect polymorphisms in taxonomic and phylogenetic studies^13^.

The conventional RAPD systems currently used for profile analysis mainly rely on visual outputs, and thus may be subjective and observer-dependent in certain cases. Thus, to improve the accuracy of RAPD profile analysis, we here propose a new method of coupling RAPD-PCR with capillary gel electrophoresis (CGE). CGE is an automated, high-throughput DNA fragment analysis method that can be readily applied for the investigation of a large number of samples. In this study, we developed a rapid CGE-light-emitting diode-induced fluorescence (LEDIF)-based method in conjunction with RAPD-PCR amplification for genotyping. To our knowledge, this combination has never been utilized for the epidemiological typing of bacteria.

Recent studies have shown that *E. anophelis* is frequently misidentified as *E. meningoseptica*^14,15^. The prevalence of *E. anophelis* infections in humans could therefore be greatly underestimated. Correctly identifying microorganisms is extremely important before subsequent molecular typing.

The main aims of our study were to establish the basis and identify effective tools for epidemiological investigations of *E. anophelis* and to provide a convenient and reliable molecular typing method for expanding the database of *E. anophelis*.

To test the performance of the novel combined method, we collected *E. anophelis* isolates from patients at our hospital (Tri-Service General Hospital TSGH) as well as at another institute, Taipei Veterans General Hospital (TVGH). Genomic DNA of the isolates was sequenced and then subjected to matrix-assisted laser desorption ionization–time of flight (MALDI-TOF) for identification confirmation at the species level, and then subjected to RAPD-PCR coupled with CGE for molecular typing. The discriminatory ability of each technique was compared based on cluster analysis and calculation of Simpson’s index of diversity (SID).

## Results

### Isolates identification using MALDI-TOF and 16 S rRNA

The MALDI-TOF Vitek MS Plus system with an amended database correctly identified all *Elizabethkingia* species. In addition to the 55 *E. anophelis* isolates, 15 *E. meningoseptica* and 10 *E. miricola*, identified with MALDI-TOF in our hospital, were chosen to determine the accuracy of bacterial identification. All isolates used in the study exhibited 100% correspondence between 16 S rRNA gene sequencing and the MALDI-TOF Vitek MS Plus system (Table 1).

**Table 1:** Comparison of *Elizabethkingia* spp. identified by 16 S rRNA gene sequencing and Vitek MS Plus MALDI-TOF system.

		Vitek MS Plus MALDI-TOF		
		*E. anophelis*	*E. miricola*	*E. meningoseptica*
**16 S rRNA Sequencing**	*E. anophelis*	55(100%)		
	*E. miricola*		10 (100%)	
	*E. meningoseptica*			15 (100%)

### Reproducibility and performance of CGE-RAPD

**Figure 1:**
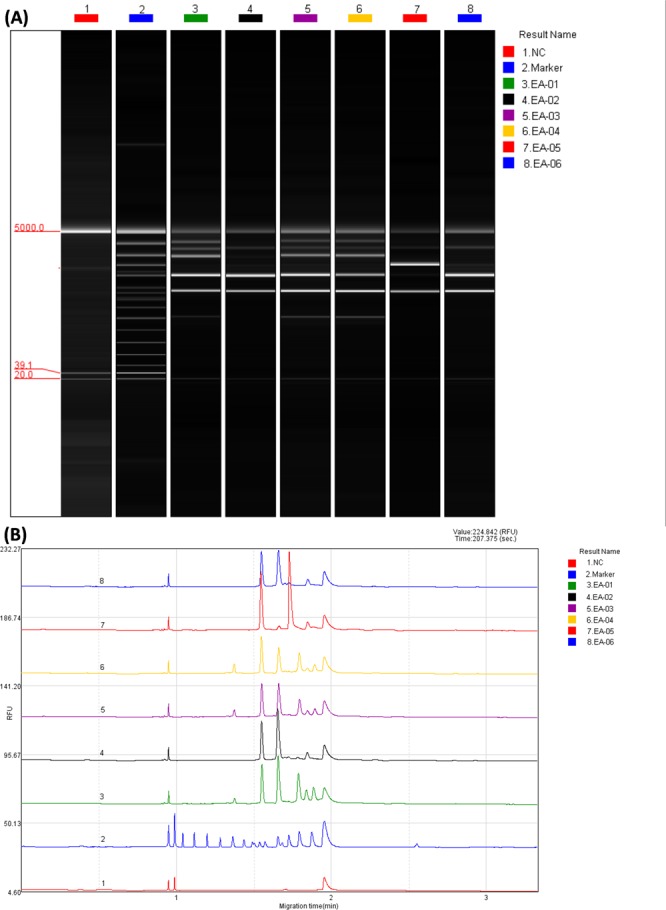
Representative RAPD results using the RAPD-1 primer. Qsep100 DNA Analyzer gel image (**B**) Qsep100 DNA Analyzer signal chart. *NC: Negative control Marker: 50–3000 bp DNA Size Marker.

After testing three primers, the primers OPA-10 and RAPD-1 were found to have potential for obtaining a clear and distinct DNA profile, with RAPD-1 showing better performance. The peaks were clearly visible by Q-Analyzer software (BiOptic Inc.). Figure 1 depicts the CGE traces of the PCR fragments generated after RAPD-PCR. A DNA sizing ladder in the range of 50–3000 bp was used for fragment size assessment, based on the signal chart and gel image. Reproducibility of RAPD was assessed using the optimized PCR mixtures and running conditions described previously^16^; all data showed identical repeatable patterns with the RAPD-1 primer. Thus, all clinical isolates of *E. anophelis* were found to be typeable (3–7 distinct bands/sample) by the RAPD protocol used in this study.

### Comparison between CGE-RPAD, PFGE and cgMLST

**Figure 2:**
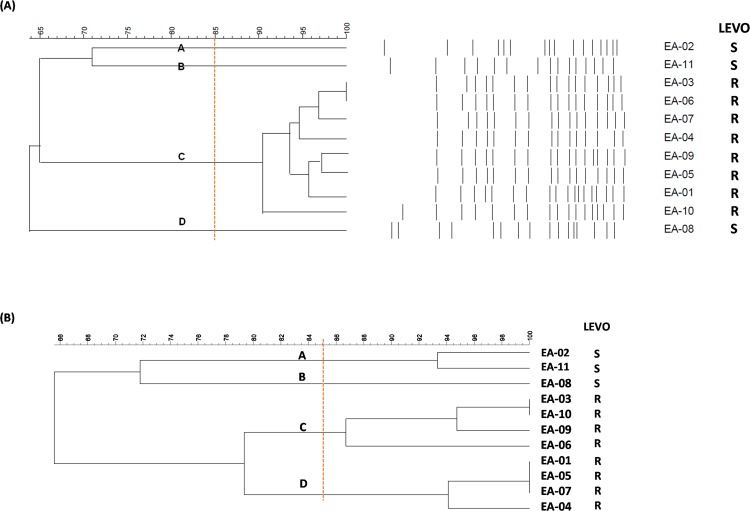
Dendrogram of 11 *Elisabethkingia anophelis* isolates clustered based on (**A**) *PFGE or (**B**) **RAPD-PCR. *Isolates were grouped according to ApaI restriction patterns using GelCompar II software. **Isolates were grouped according to RAPD PCR banding patterns with GelCompar II software.

Pulsed-field gel electrophoresis (PFGE) has been considered the ‘gold standard’ among molecular typing methods for a variety of clinically important bacteria. During 2017, 11 strains of *E. anophelis* were isolated from blood specimens in our hospitals to investigate epidemiological relatedness. PFGE data were obtained with Dr. Kuo’s team’s generous assistance. To test the discriminatory ability of our RAPD method for *E. anophelis* molecular typing, we compared molecular typing results using the two methods: RAPD and PFGE (Fig. 2). The clustering result showed good consistency between the two methods (Table 2). All 11 *E. anophelis* isolates were clustered into four pulsotypes (A~D); however, there were still different clustering results; e.g., EA-02, and EA-11 were clustered into two groups by FPGE but were viewed as the same group by RAPD. Another discrepancy was found wherein EA-01, 03, 04, 05, 06, 07, 09, and 10 were classified into the same group by PFGE but clustered into two groups by RAPD. These results may be attributed to the characteristics of the different methodologies. To verify whether the cluster patterns obtained by these two molecular typing methods were both accurate, we examined the 11 *E. anophelis* isolates from our hospital by determining the minimum inhibitory concentration (MIC) of quinolone using broth microdilution (3 susceptible isolates, 8 resistant isolates) (Table 3). Three quinolone (EA-02, EA-08, EA-11)-susceptible isolates *E. anophelis* were divided into different clusters and compared with eight quinolone-resistant isolates by both FPGE and RAPD (Fig. 2).

**Table 2:** Comparison of clustering patterns of 11 *E. anophelis* isolates by PFGE or RAPD.

Cluster by	PFGE^a^	Cluster by	RAPD^b^
A	EA-02	EA-02, 11	A
B	EA-11	EA-08	B
C	EA-01, 03, 04, 05, 06, 07, 09, 10	EA-03, 06, 09, 10	C
D	EA-08	EA-01, 04, 05, 07	D

^*^PFGE and RAPD: clustering cutoff value by 85% similarity.

^a^PFGE: pulsed-field gel electrophoresis. ^b^RAPD: Random amplified polymorphic DNA.

**Table 3:** Ciprofloxacin/levofloxacin MIC values of 11 *E. anophelis* isolates.

Species	Number of isolates	BMD MIC (µg/ml)		susceptibility
		CIP	LEVO	
*Elizabethkingia anophelis*	EA-01	64	64	R
	EA-02	1	0.25	S
	EA-03	32	64	R
	EA-04	64	64	R
	EA-05	32	32	R
	EA-06	32	32	R
	EA-07	32	32	R
	EA-08	2	1	S
	EA-09	32	32	R
	EA-10	32	64	R
	EA-11	2	1	S

CIP, ciprofloxacin; LEVO, levofloxacin, S, susceptible; R, resistant

BMD (broth microdilution) susceptibility (≤value) and resistance (≥value) breakpoints defined by Clinical and Laboratory Standards Institute: 2 µg/ml and 8 µg/ml for levofloxacin, 1 µg/ml and 4 µg/ml for ciprofloxacin.

Further confirmation study of performance of CGE-RAPD was conducted with another 13 *E. anophelis* isolates, and the RAPD clustering results were also consistent with PFGE (Supplementary Figs 1, 2).

Another well-established microbial typing method is MLST. However, there is no standard MLST (multilocus sequence typing) molecular typing method database about *E.anophelis*. With the advantage NGS techniques, a study by Breurec *et al*.^17^ constructed a standardized MLST composed of high-resolution core genome data (cgMLST) consisting of 1546 alleles. To verify our RAPD method compared with cgMLST, we used four *E. anophelis* isolates whole genomes and phylogenetic tree was constructed to infer the relationship among these genomes using the concatenated sequences of core proteins identified from the core genome analysis. The tree separated the four *E.anophelis* genomes into four distinct branches consistent with our RAPD clustering results (Supplementary Fig. 3).

### Multicentre molecular epidemiology with CGE-RAPD

**Figure 3:**
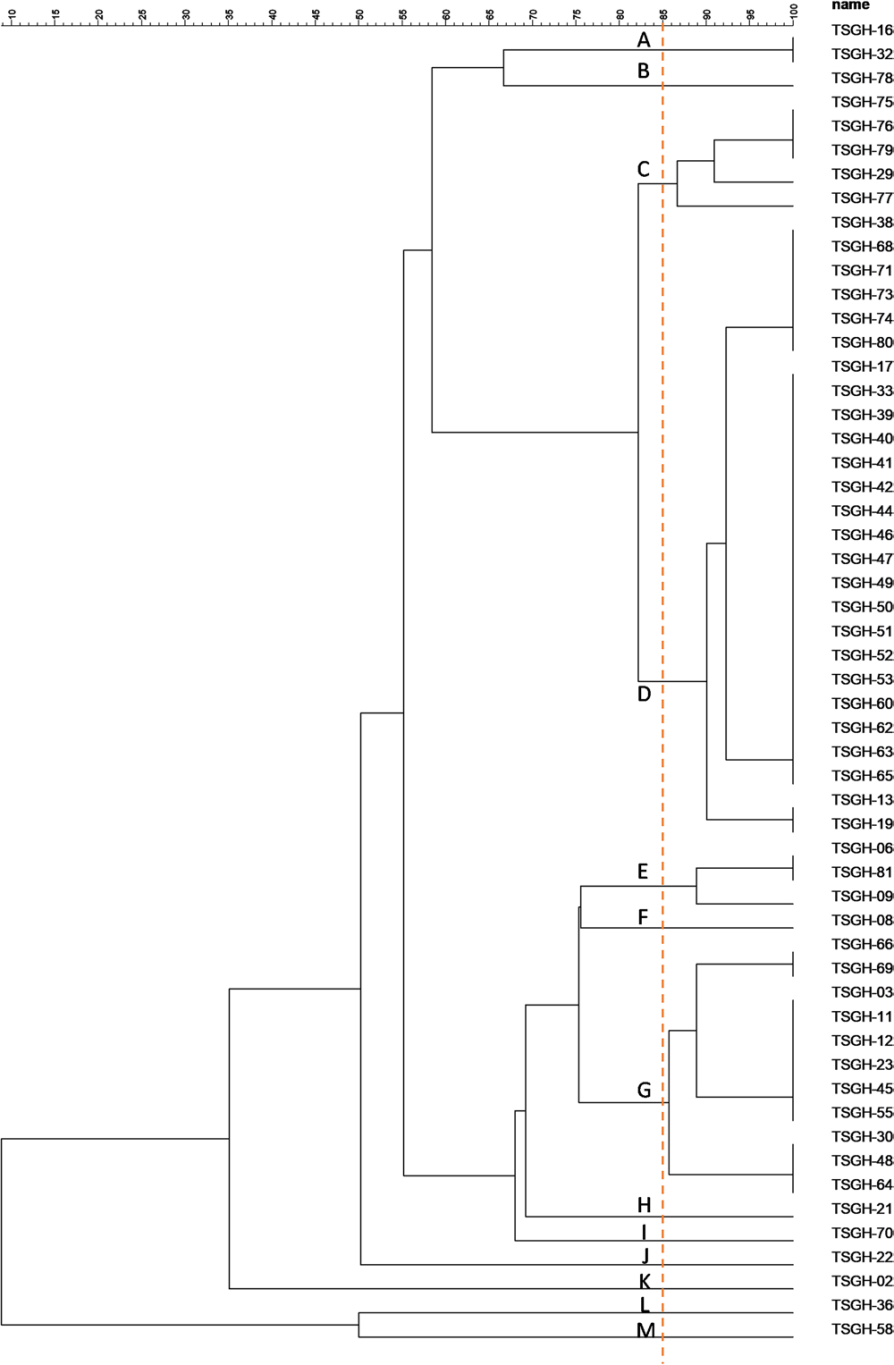
RAPD dendrogram of 55 *Elisabethkingia anophelis* isolates from our hospital (TSGH). 13 different pulsotypes (A~M) are delineated at a cut-off similarity level of 85%.

The epidemiological relatedness of the 55 isolates from our hospital (TSGH) was determined by RAPD (Fig. 3). A dendrogram was generated by the RAPD banding pattern, and the clustering dendrogram was performed with GelCompar II software. Dice similarity coefficients were calculated and clustering was performed by unweighted pair group mean association. With a similarity coefficient of 85% as a cut-off value, 13 major clusters were found, each represented by different members, among the isolates from our hospital. The SID value of our RAPD technique was above 0.9 (Table 4), indicating a relatively high level of discrimination.

**Table 4:** Number of genotypes and Simpson’s index of diversity (SID) according to the RAPD genotyping scheme.

Primer	#partitions	SID	CI (95%)	CINA (95%)
RAPD-1	13	0.937	0.934–0.940	0.931–0.943

CI: confidence interval CINA: Non-Approximated Confidence Interval.

**Figure 4:**
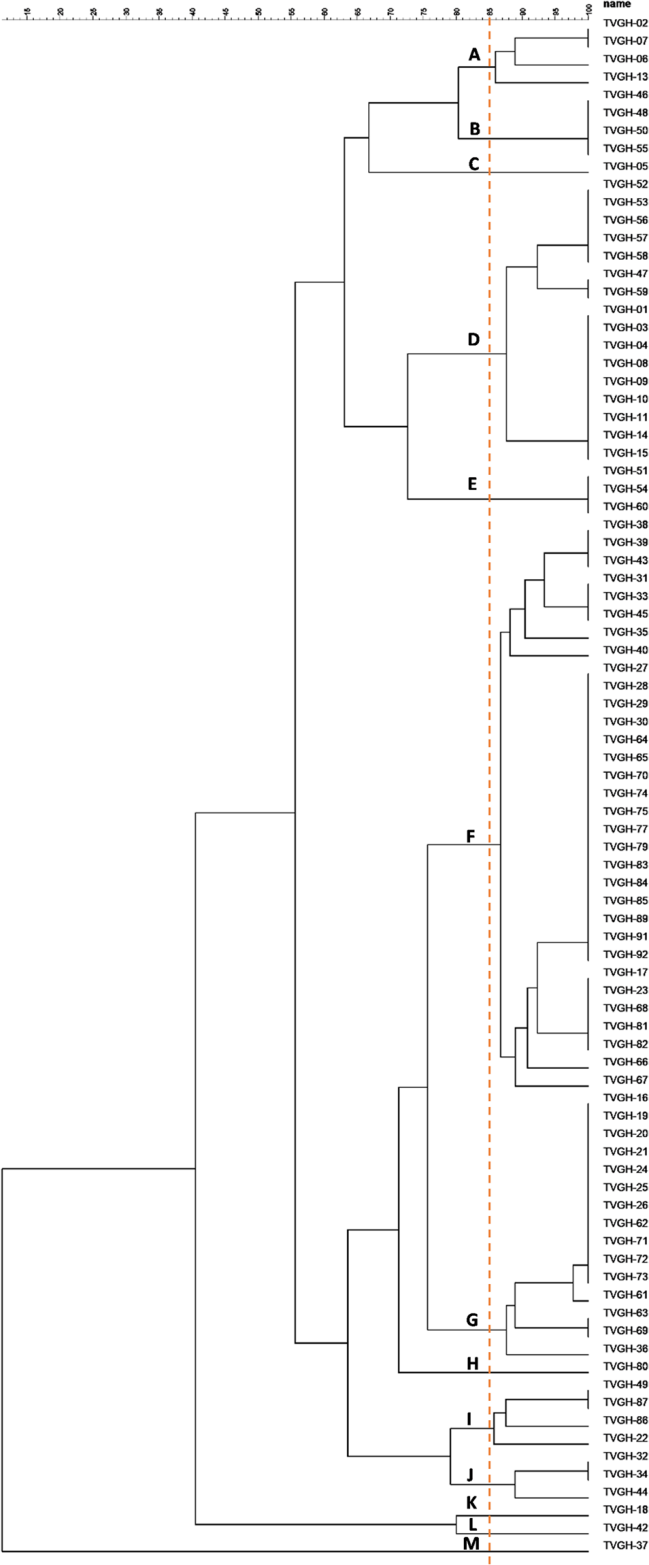
RAPD dendrogram of 86 *Elisabethkingia anophelis* isolates from another medical center(TVGH). 13 different pulsotypes (A~M) are delineated at a cut-off similarity level of 85%.

**Figure 5:**
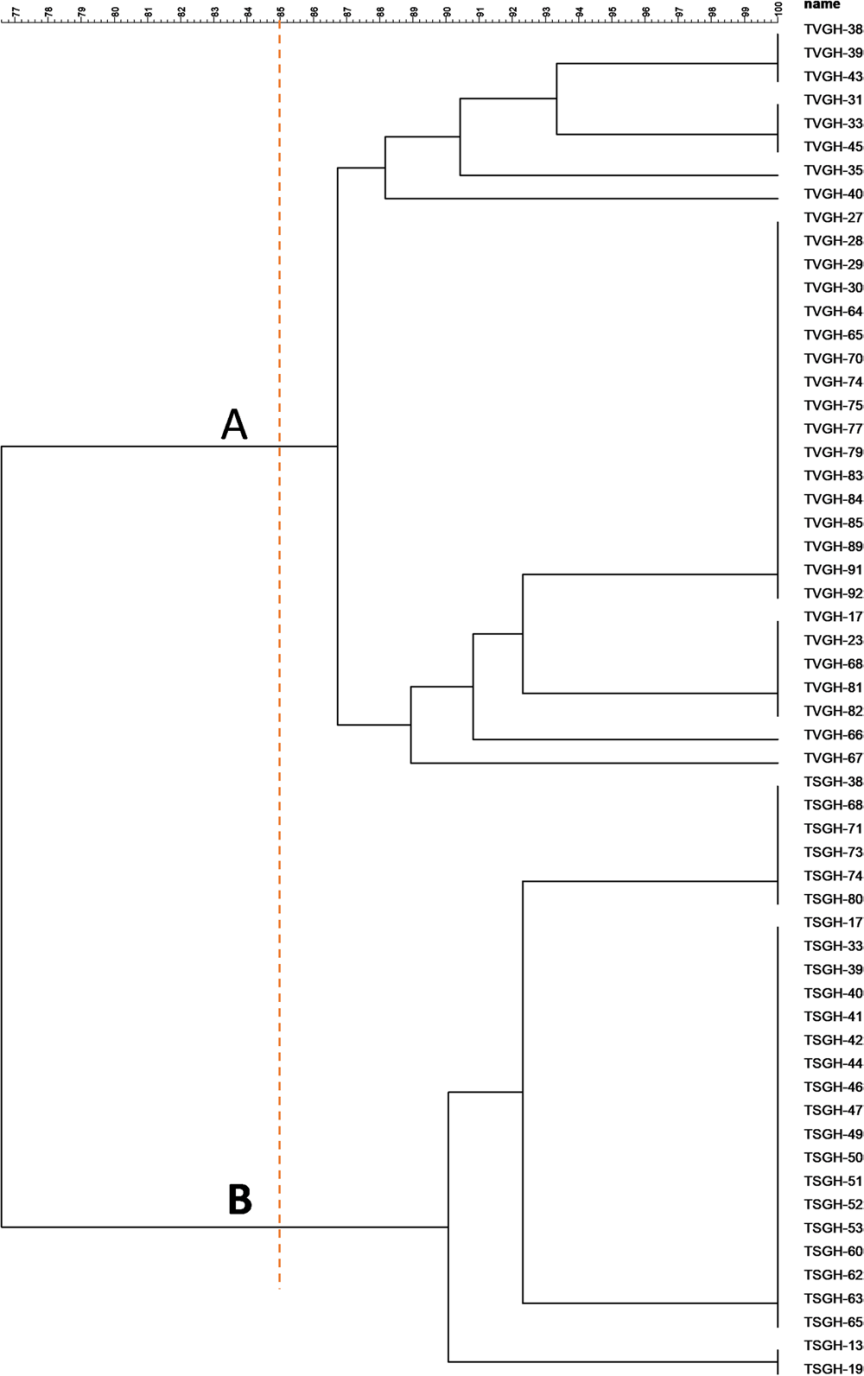
Comparison of the clustering results between the *Elisabethkingia anophelis* isolates from two hospitals (TSGH and TVGH). The cutoff value for cluster delineation was 85% similarity.

To address the typing discriminatory power, we also used RAPD as a molecular typing method for 86 *E. anophelis* isolates from another medical centre in northern Taiwan (isolates denoted as TVGH); 13 pulsotypes were obtained with a similarity cutoff coefficient of 85% (Fig. 4). The aforementioned results indeed authenticated the RAPD molecular typing method in *E. anophelis* isolates and could be applied at different health care institutions with healthcare-associated infection strains. We were also interested in the major cluster between the two medical centres because these two hospitals are in the same geographic location, i.e., northern Taiwan. Here, we selected 26 isolates from our hospital (denoted as TSGH) clustering in the same group and 32 isolates from the other hospital (denoted as TVGH) clustering in one group. Further molecular typing of the major clusters from the two hospital sources demonstrated that the isolates from the two hospitals formed two distinct clusters (Fig. 5).

## Discussion

Typing methods for discriminating different bacterial isolates of the same species are essential epidemiological tools in infection prevention and control. The recent development of new methods for examining the relatedness of isolates at the molecular level has revolutionized the ability to differentiate among bacterial types and subtypes^13^. Although the emergence of bench-top sequencers using next-generation sequencing technology has made bacterial whole-genome sequencing (WGS) feasible, this technology is still too laborious and time-consuming for widespread application to obtain useful data in routine surveillance^18^. Since the invention of MLST in 1998, this technique has been confirmed to be highly reproducible, objective, and with sufficient discriminatory power for the molecular typing of bacteria, and can be performed easily by different laboratories for the typing of strains. However, some studies have demonstrated that the phylogeny inferred from MLST data failed to accurately represent the genome-based phylogeny for the same bacterial species^11,19^. A wide variety of bacterial typing systems are currently in use that vary greatly with respect to the effort required, cost, reliability, and ability to discriminate among bacterial strains. Thus, choosing an appropriate bacterial typing technique for epidemiologic studies is essential for epidemiologic investigations^11^.

Fingerprinting techniques are important tools in understanding the genetic diversity of *E. anophelis* and for epidemiologic monitoring. Several molecular fingerprinting techniques, including PFGE, MLST, and WGS, have been utilized to date^5,7,17,20^. However, these studies generally use a small number of strains to investigate phylogenetic relationships or outbreak-related situations. In the present study, molecular typing of *E. anophelis* with our species-specific primer allowed for the correct amplification of all the *E. anophelis* isolates tested.

The general criteria for evaluating typing systems include factors such as reproducibility, typeability, discriminatory power, ease of interpretation, and ease of performance.

Fingerprinting techniques are important tools for understanding the genetic diversity of *E. anophelis* and the epidemiologic relatedness. A variety of molecular fingerprinting techniques such as PFGE, MLST (multilocus sequence typing), and WGS have been utilized in the molecular typing of *E. anophelis*^10^. Lau *et al*. used PFGE as a molecular typing method^5,7^. whereas Breurec *et al*. constructed a standardized MLST composed of high-resolution core genome data (cgMLST) for typing *E. anophelis*^17^. WGS has also been used to compare single nucleotide polymorphisms (SNPs) between related and unrelated genomes^20^.

The aforementioned molecular typing methods suffer from several limitations such as being technically demanding (for PFGE, MLST, and WGS), labour-intensive and time-consuming (for PFGE, MLST, and WGS), and lacking the resolution power to distinguish bands of nearly identical size (for PFGE). Moreover, analysis of PFGE results is prone to some subjectivity. Lin *et al*. found that one isolate was resistant to restriction enzyme digestion; hence, the isolate failed molecular typing by PFGE^21^.

PCR-based methods such as RAPD have the advantage of being simpler and less labour-intensive compared to PFGE, and thus more amenable to high-throughput screening in a microbiology laboratory. PCR assays can also be developed to rapidly diagnose specific, problematic clonal strains. To our knowledge, this study represents the first assessment of the reproducibility of molecular typing techniques for *E. anophelis* isolates obtained from bacteraemia patients. One of the most important strengths of this study is its generalizability, along with the relatively large sample size. Use of such a reproducible RAPD-PCR method would allow for efficient analyses of *E. anophelis* genetic diversity at different laboratories or at different time periods within the same laboratories. In our experiments, the RAPD-1 primer showed the most reproducible performance, and could also generate identical profiles of template DNA over a range of different time courses (data not shown).

We further compared the properties of our genotyping scheme for the phylogenetic analysis of *E. anophelis*. Here, we used a rapid CGE-based method in conjunction with RAPD-PCR amplification for genotyping to more efficiently dissect subtle genomic differences, especially among very closely related isolates. Indeed, we demonstrated a high level of discrimination in genotyping *E. anophelis*. Thus, our RAPD-PCR method coupled with CGE has potential to be a cost-efficient, high-throughput screening test to investigate possible nosocomial strains and the genetic diversity among *E. anophelis* clinical isolates.

## Materials and Methods

### Bacterial isolates

Clinical isolates (55 and 86) were collected from bacterial cultures from the Tri-Service General Hospital (TSGH) and the Taipei Veterans General Hospital (TVGH), respectively, two tertiary care centres in northern Taiwan, between 2013 and 2017. All isolates or extracted DNA samples were derived from blood cultures. Species were initially identified using the Vitek MS system with the IVD 3.0 database (bioMérieux, Mercy l’Etoile, France). Isolates identified as *Elizabethkingia* spp. were kept frozen until use in this study.

### DNA extraction

Genomic DNA was isolated using a previously reported protocol^22^. In brief, genomic DNA was extracted from a single colony of the isolates from the two hospitals. The concentrations of the purified genomic DNA were measured at 260 nm and the purity was estimated by measuring the ratio of the optical density at 260 nm and 280 nm with a Picodrop (Picodrop, Hinxton, UK) spectrophotometer. DNA samples were stored at −20 °C until RAPD was performed. The quality of the DNA samples was checked on 2% agarose gels.

### 16 S rRNA identification

The microbial identification accuracy was verified with 16 S rRNA sequencing using a pair of species-specific primers: 27 F (5′-AGAGTTTGATCMTGGCTCAG-3′) and 1492 R (5′-GGYTACCTTGTTACGACTT-3′). PCR conditions were described previously^23^. The expected amplicon length was 1488 bp. All amplified DNA fragments were sequenced using the ABI 3500 system with Big-Dye Terminator v3.1 Cycle Sequencing Kit (ABI Prism, Foster City, CA, USA) and the DNA sequencing data were searched using the Basic Local Alignment Search Tool (BLAST) in the National Center for Biotechnology Information database. The species was considered identified if the isolate possessed 99.5% 16 S rRNA sequence identity to the type strain.

### MALDI-TOF identification

All clinical isolates were further identified by the VITEK MS Plus (bioMérieux) MALDI-TOF system. The spectra were acquired in linear positive-ion mode at a laser frequency of 50 Hz in the m/z range of 2,000 to 20,000 Da. The *Escherichia coli* reference strain ATCC 8739 was used for instrument calibration on each section of the target slide according to the manufacturer’s instructions. Spectral data of all clinical isolates were collected and analysed with Saramis Premium software. Subsequently, the spectra from the *E. anophelis*, *E. miricola*, and *E. meningoseptica* isolates were imported into the RUO Saramis database. Under the spectral taxonomy tree, new folders of species were established under the genus *Elizabethkingia*, and the imported spectra were added into the respective folders. SuperSpectra of *E. anophelis, E. miricola*, and *E. meningoseptica* were created with Saramis Premium software, and the criterion of the frequency in each peak was set to >60%, which was reduced for common peaks between species.

### Antimicrobial susceptibility

Minimum inhibitory concentrations (MICs) of ciprofloxacin and levofloxacin were established by the microbroth dilution method recommended by the Clinical and Laboratory Standards Institute (CLSI). The breakpoints proposed by CLSI were used for ciprofloxacin (susceptibility, 1 μg/mL; resistant, 4 μg/mL) and levofloxacin (susceptibility, 2 μg/mL; resistant, 8 μg/mL).

### RAPD-PCR

RAPD-PCR was performed with the OPA-10 primer (5′-GTGATCGCAG-3′), OPB-15 primer (5′-GGAGGGTGTT-3′), and RAPD-1 primer (5′-GTCGATGTCG-3′), as previously described by Hsueh *et al*.^24^ and Chiu *et al*.^25^. Oligonucleotides were commercially synthesized by Genomics (Taiwan). The primers were resuspended in Tris-ethylenediaminetetraacetic acid buffer, stored at −20 °C, and 10 μM/μl working solutions were prepared for use in PCR.

The reaction mixture (25 μl) contained 10 mM Tris–HCl (pH 7.5), 50 mM KCl, 2.5 mM MgCl_2_, 0.5 mM spermidine, 0.1 mM dNTPs, 15 pmol of the RAPD primer, 50 ng genomic DNA, and 0.8 U of DyNAzyme II DNA Polymerase (ABI, Thermo Fisher Scientific). For every sample, each RAPD reaction was performed at least twice for each DNA extract. Amplification was carried out in a ProFlex PCR system (Applied Biosystems) thermal cycler with one initial denaturation step of 5 min at 95 °C; 40 cycles of a denaturing step of 1 min at 94 °C, followed by an annealing step of 1 min at 36 °C, and an extension step of 2 min at 72 °C; and a final elongation step at 72 °C for 8 min. The RAPD reaction products were stored at −20 °C until further analysis.

### CGE-based analysis

After PCR amplification, the products were analysed on Qsep100 DNA Analyzer (Qsep-10, Bioptic, Taiwan). A disposable pen-shaped cartridge was inserted and other running buffers were loaded according to the manufacturer’s instructions^26^.

PCR fragments were applied into a miniaturized single-channel capillary cartridge of the Qsep100 DNA-CE unit with separation buffer. The run was performed using a high-resolution cartridge with a sample injection protocol of 8 kV for 10 s and separation at 5 kV for 300 s. The DNA alignment markers (20 bp, 1.442 ng/μl and 5000 bp, 1.852 ng/μl) and the DNA size marker (50–3000 bp, 10.5 ng/μl) were obtained from BiOptic. Samples peaks were visualized using Q-Analyzer software (BiOptic Inc.).

### PFGE analysis

The E. *anophelis* isolates were characterized by PFGE using a CHEF Mapper XA system (Bio-Rad, Hercules, CA, USA) with the restriction endonuclease *Xba*I as described previously^5^. The bacteria were suspended in 3 ml cell suspension buffer (20 mM Tris and 40 mM EDTA; pH 8.0) with an optical density at 610 nm (OD_610_) of 1.6–1.8. Agar plugs (200 ml bacterial aliquot, 100 mg proteinase K, and 200 ml of 1% agarose) were prepared and treated with cell lysis buffer (50 mM Tris, 50 mM EDTA, 1% *N*-lauroylsarcosine, and 500 mg proteinase K; pH 8.0), and then the DNA was digested with *Xba*I at 37 °C for 4 h. PFGE was performed using the CHEFDR III system (Bio-Rad), and the data were analysed using GelCompar II software (GelCompar II, Applied Maths NV, Belgium). The Dice similarity coefficients were calculated and clustering was performed by unweighted pair group mean association (UPGMA).

### RAPD-PCR typing results and statistical analysis

Isolates were categorized as identical, similar, or unrelated according to their PCR banding patterns. The data were analysed using GelCompar II software (Applied Maths NV, Sint-Martens-Latem, Belgium). Dice similarity coefficients were calculated and clustering was performed by unweighted pair group mean association (UPGMA). The index of diversity of our typing schemes was determined with an online tool (http://www.comparingpartitions.info/; accessed 10/10/2018). The discriminatory ability of the different techniques described above was evaluated using the SID (with 95% confidence intervals) as described previously^27^.

### Genome characterization of *E. anophelis*

The DNA of the *E. anophelis* isolate was prepared using a Qiagen genomic DNA purification kit according to the manufacturer’s instructions (Qiagen, Hilden, Germany), and the genome was sequenced using an Illumina MiSeq. 2000 sequencing platform (Illumina, CA, USA). The short reads were assembled and optimized according to paired-end and overlap relationships via mapping reads to the contig using SOAP de novo. Subsystem technology prokaryotic genome annotations were based on Rapid Annotation using Subsystem Technology (RAST).

### Core genome multilocus sequence typing (MLST) and phylogenetic analysis

CgMLST typing method with 32 isolates of *Elizabethkingia* species isolates from NCBI database was described previously^17^. These loci together constitute a cgMLST scheme useful for genotyping of *E. meningoseptica*, *E. anophelis*, and *E. miricola*. Phylogenetic tree was constructed to infer the relationship among these genomes using the concatenated sequences of core proteins identified from the core genome analysis using Molecular Evolutionary Genetics Analysis (MEGA) version X^28^.

### Ethics

This study was approved by the Tri-Service General Hospital Institutional Review Board (TSGH IRB 2-107-05-035), registered on 23 April 2018, and the requirement of patient consent was waived.

## Supplementary information

Supplementary information

## Data Availability

All data generated or analysed during this study are included in this published article (and its Supplementary Information files).
