# Supplementary material for: Multicentre MDR Elizabethkingia anophelis isolates: Novel random amplified polymorphic DNA with capillary electrophoresis systems to rapid molecular typing compared to genomic epidemiology analysis
Source: Sci Rep. 2019 Feb 12;9:1806. doi: 10.1038/s41598-019-38819-w (PMC6372666; doi:10.1038/s41598-019-38819-w)

Supplementary information

Multicentre MDR *Elizabethkingia anophelis* isolates: Novel random amplified polymorphic DNA with capillary electrophoresis systems to rapid molecular typing compared to genomic epidemiology analysis

Ming-Jr Jian[1,2], Cherng-Lih Perng[1,2], Jun-Ren Sun[3], Yun-Hsiang Cheng[1,2], Hsing-Yi Chung[2], Yu-Hsuan Cheng[2], Shih-Yi Lee[4], Shu-Chen Kuo[5], Hung-Sheng Shang[1,2]

[1]Graduate Institute of Medical Science, National Defense Medical Center, Taipei, Taiwan

[2]Division of Clinical Pathology, Department of Pathology, Tri-Service General Hospital, National Defense Medical Center, Taipei, Taiwan

[3]Institute of Preventive Medicine, National Defense Medical Center, Taipei, Taiwan

[4]Division of Clinical Microbiology, Department of Pathology and Laboratory Medicine, Taipei Veterans General Hospital, Taipei, Taiwan

[5]National Institute of Infectious Diseases and Vaccinology, National Health Research Institutes, Zhunan, Taiwan

***Corresponding authors:**

Hung-Sheng Shang, e-mail: iamkeith001@gmail.com

National Defense Medical Center, 325, Section 2, Cheng-Kung Road, Neihu 114, Taipei, Taiwan, Republic of China

Supplementary data legends

Supplementary Figure 1. Dendrogram of another 13 *E.anophelis* isolates by PFGE or RAPD

Supplementary Figure 2. Comparison of clustering patterns of 13 *E. anophelis* isolates by PFGE or RAPD.

Supplementary Figure 3. Comparison of the clustering results of four *E.anophelis* isolates between RAPD and cgMLST.

(A) PFGE

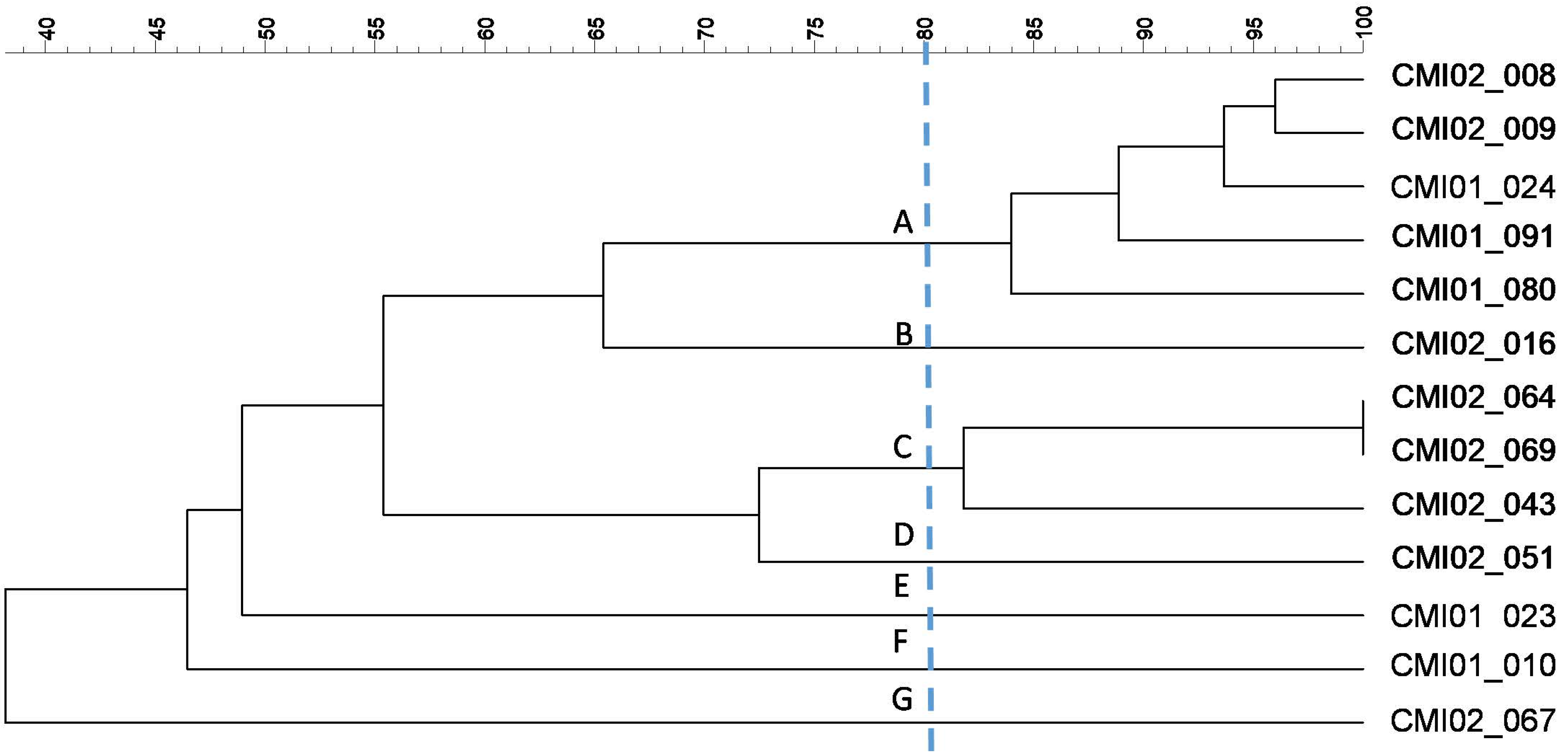

(B) RAPD-CGE

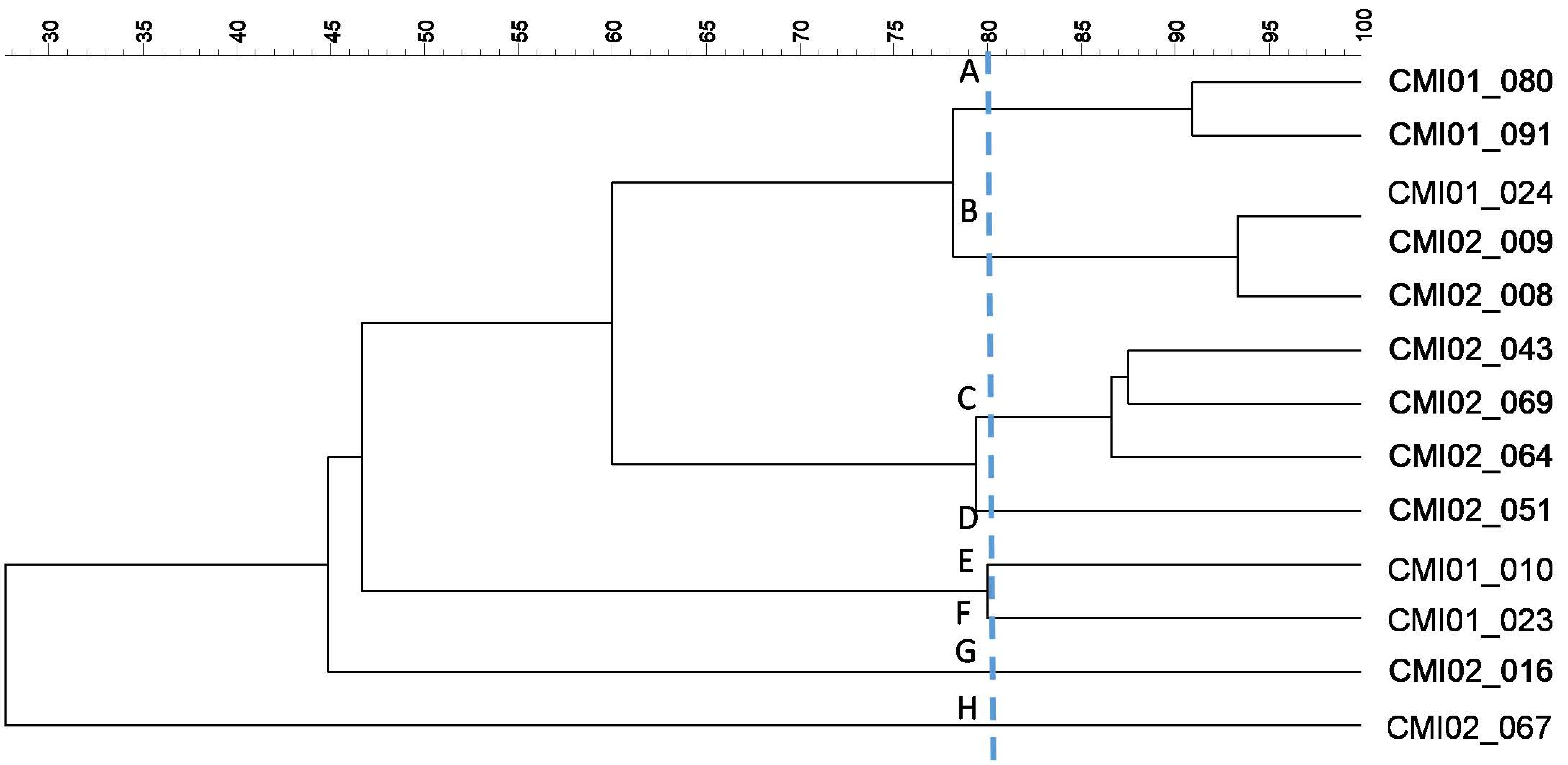

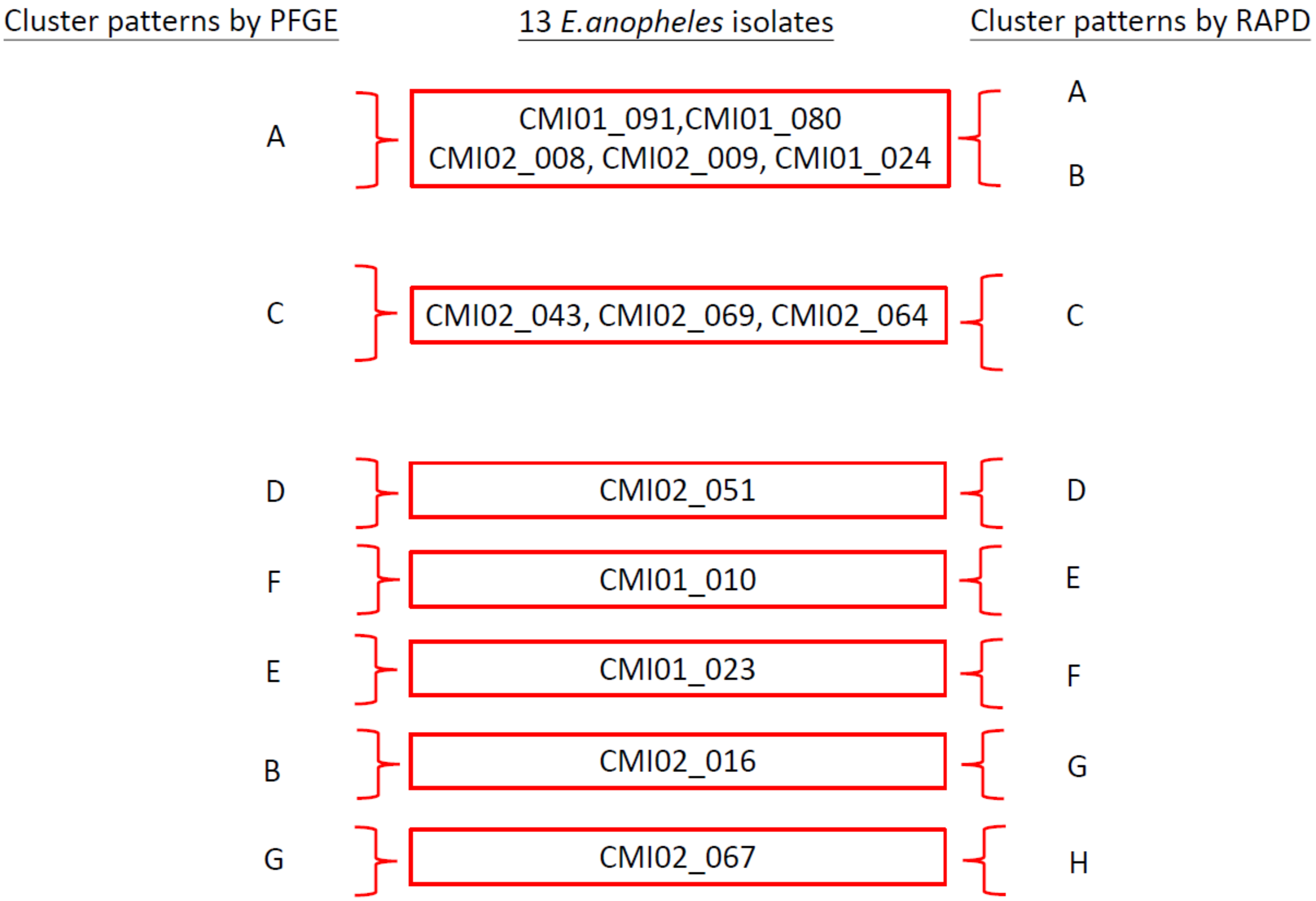
Cluster patterns by PFGE
13 *E.anopheles* isolates
Cluster patterns by RAPD
A
CMI01_091,CMI01_080
CMI02_008, CMI02_009, CMI01_024
A
B
C
CMI02_043, CMI02_069, CMI02_064
C
D
CMI02_051
D
F
CMI01_010
E
E
CMI01_023
F
B
CMI02_016
G
G
CMI02_067
H

(A) RAPD

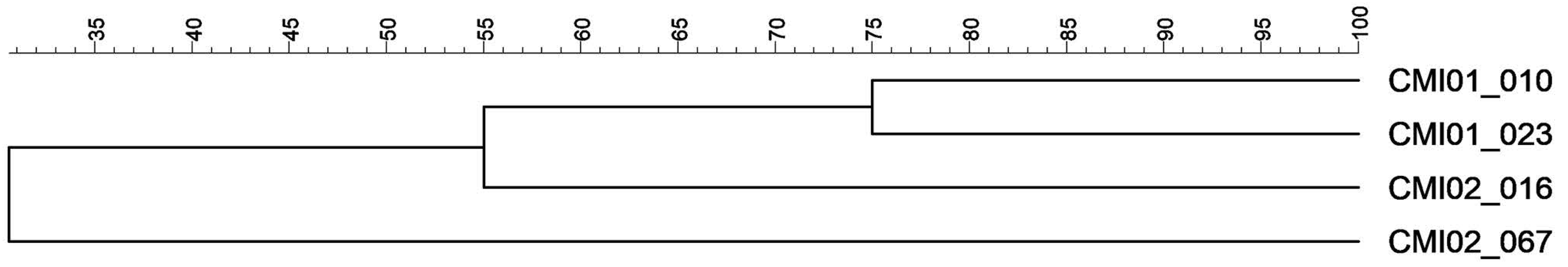

(B) cgMLST

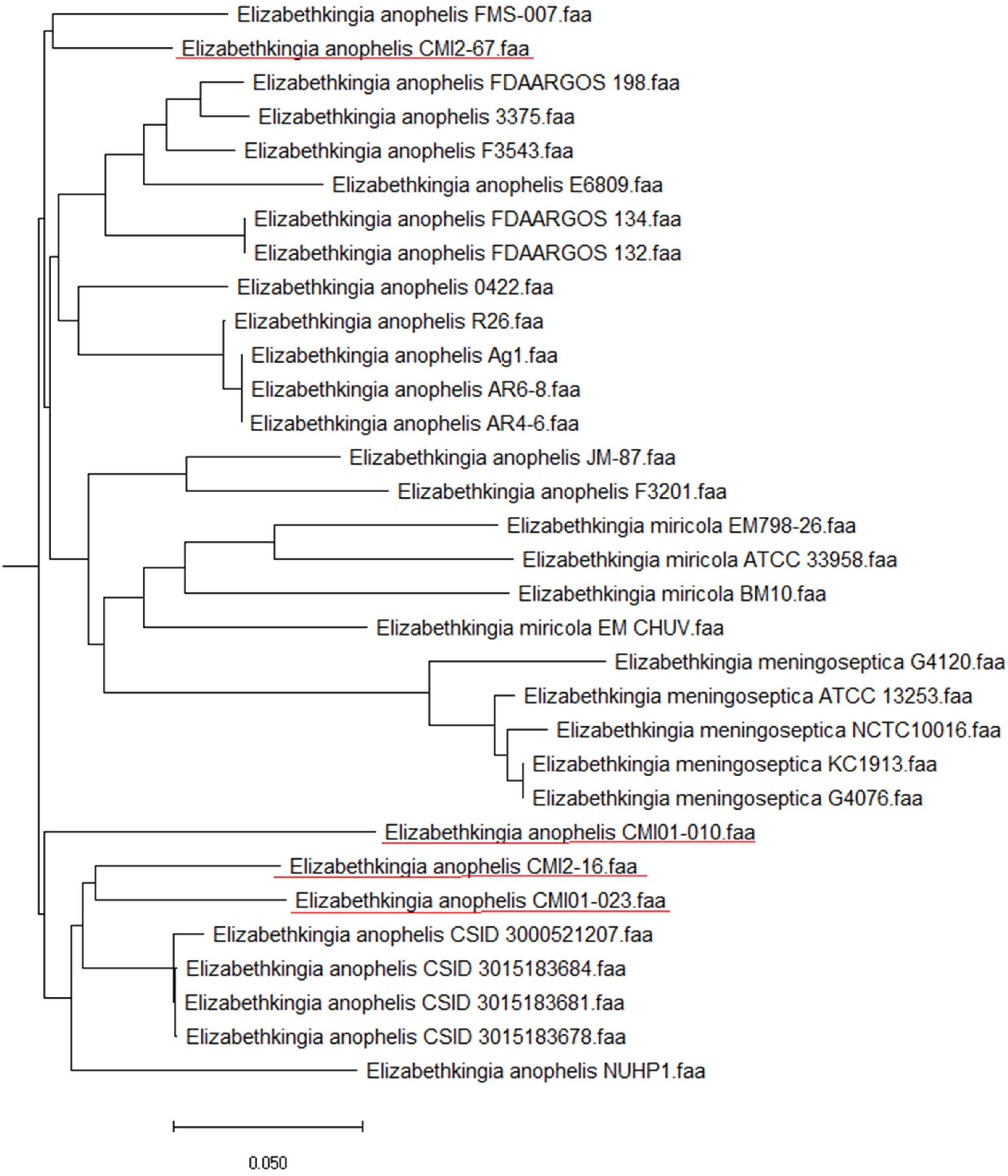

Supplement: Supplementary file 1 — Supplementary information [file 41598_2019_38819_MOESM1_ESM.pdf]
